# Effects of nutritional intake on disease severity in children with sickle cell disease

**DOI:** 10.1186/s12937-016-0159-8

**Published:** 2016-04-30

**Authors:** Valentina Mandese, Francesca Marotti, Luca Bedetti, Elena Bigi, Giovanni Palazzi, Lorenzo Iughetti

**Affiliations:** 1 School of Pediatrics, Department of Medical and Surgical Sciences for Mothers, Children and Adults, University of Modena and Reggio Emilia, Modena, 41124 Italy; 2 Pediatric Unit, Department of Medical and Surgical Sciences for Mothers, Children and Adults, University of Modena and Reggio Emilia, Via del Pozzo 71, Modena, 41124 Italy; 3 Oncology and Hematology Pediatric Unit, Department for Maternal and Child Integrated Activities, University Hospital of Modena, Modena, 41124 Italy; 4 Department of Medical and Surgical Sciences for Mothers, Children and Adults, University of Modena, and Reggio Emilia, Via del Pozzo 71, Modena, 41124 Italy

**Keywords:** Diet, Macronutrient, Micronutrient, Nutritional intake, Disease severity, Sickle cell disease

## Abstract

**Background:**

Children with Sickle Cell Disease (SCD) may show growth failure in comparison to healthy peers. Many factors as hematological status, endocrine and/or metabolic dysfunction, and nutritional status, may play an important role in growth failure. The aim of this study was to assess whether impaired growth and nutritional intake can affect SCD severity during childhood.

**Methods:**

We conducted an observational study on children with SCD referring to our clinic for routine follow-up visits in a 6-month period. We collected information on weight, height and body mass index (BMI) and calculated their respective standardized scores (z). The nutritional intake was assessed through the last 24-h recall intake of total calories, macro- (proteins, lipids, carbohydrates) and micronutrients (calcium, iron, phosphorus, vitamins B1, PP, A, C, B2). Disease severity was assessed through total hemoglobin (Hb) and fetal hemoglobin (HbF), and lactic dehydrogenase (LDH) levels, and through the total number and days of hospitalizations, as well as the lifetime episodes of acute chest syndrome (ACS).

**Results:**

Twenty nine children (14 males, 15 females) with SCD were enrolled; their mean age was 9.95 years (SD 3.50, min 3.72, max 17.18). Z-weight and z-BMI were significantly directly related to total Hb. Food intake resulted significantly unbalanced in terms of total calorie intake, macro- and micronutrients, especially calcium, iron, vitamin B1 and C. Low intake of calcium and vitamin B1 were significantly inversely correlated with number and days of hospitalizations per year. Protein, lipid, phosphorus, and vitamin PP intakes resulted adequate but were inversely correlated with number and days of hospitalizations. Carbohydrate, lipid, iron, phosphorus, vitamins B1 and B2 intakes were significantly inversely correlated to HbF levels.

**Conclusions:**

This study showed that, in our population, inadequate nutritional intake, weight and BMI have a significant impact on SCD severity indices.

## Background

SCD is a life-threatening genetic disorder characterized by chronic hemolytic anemia, vascular injury and organ dysfunction. Over the last few decades there has been a significant improvement in the management of patients with SCD. However, SCD may affect physical growth during childhood and early adolescence, and children with this condition are often leaner and shorter than their healthy peers [1]. The prevalence of low weight in American children with SCD is 41 % with moderate and 25 % with severe under-nutrition, with a prevalence of wasting of 11 % [2, 3]. Growth failure in SCD seems to depend on multiple factors: hematologic and cardiovascular status, endocrine and metabolic functions, and nutritional status [4]. Patients with SCD have a series of micronutrient deficiencies: vitamins A, B2, B6, B12, C, D and E, folic acid, iron, calcium, magnesium and zinc [5]. It has been suggested that a better nutrition could improve body composition, especially lean mass, and have a positive impact on SCD morbidity and mortality [6]. Folic acid is widely administered to children with SCD, although its optimal daily requirement has not been established. Other nutrients such as zinc, glutamine, L-arginine and antioxidants could provide important therapeutic benefit in children with SCD [5].

Although growth failure and under-nutrition are common, the underlying mechanisms have not been well studied and the precise roles of intrinsic and extrinsic factors are unclear. Inadequate food intake or increased demands associated with higher energy expenditure and requirements could be both responsible [6].

The aim of this study was to assess whether impaired growth and nutritional intake may affect SCD severity in children.

## Methods

We conducted an observational study involving children with SCD referring to our clinic during a 6-month period for their routine follow-up visits. Inclusion criteria were age between 6 months and 18 years and SCD diagnosis. Patients with comorbidities, lost to follow-up or transferred to other centers were excluded. All patients underwent a complete clinical history and physical examination including anthropometric measurements that were performed by fully-trained examiners according to the Anthropometric Standardization Reference Manual [7]. Height was measured to the nearest 0.1-cm with a calibrated wall mounted stadiometer (Harpenden, Crymych; UK) and weight was measured to the nearest 0.1-kg with a calibrated scale. BMI was calculated by dividing weight in kg by height squared (m2). Z-height, z-weight and z-BMI were calculated using the British 1990 Growth Reference Data, by using the site http://www.paediatrics.co.uk/nicu/growth-charts.

As indices of severity we considered average of total Hb, HbF and LDH over the last 6 months, as well as the average number of hospitalizations and days of hospitalization per year, and the total number of lifetime ACS episodes.

Nutritional intake was assessed with a last 24-h food recall and through the average weekly consumption of major food categories. The major food categories examined were: meats, fish, eggs, cheese, milk, fruits, vegetables, grains, legumes, and sugary beverages. The amount of food intake was assessed using a photographic atlas of food portions corresponding to precise quantities (milliliters, grams). To avoid biases due to an unusual type of diet in the 24-h recall diary, we compared it to the weekly consumption of major food categories.

The estimated total calories, macronutrients (proteins, carbohydrates, lipids) and micronutrients (calcium, iron, phosphorus, vitamins B1, B2, PP, A and C) daily intakes were calculated for each child using the software “La dieta del Sole” (M. Riva, M. Paolazzi, G. Orlandi, Coop) used by the Italian National Research Institute for Food and Nutrition. For each child we also calculated, depending on age, gender, height, weight, and physical activity, the daily requirement of calories, macro- and micronutrients according to LARN (Level Assumption Recommended Nutrients-SINU, 3rd edition).

Finally we compared calorie and macro- and micronutrients daily requirements with the actual intake: the results were showed as percentage.

All results are reported as the mean (SD, min, max). The variables examined in the study were compared with Student’s *t*-test, analysis of variance and multiple regressions. All continuous variables that didn’t show a normal Gaussian distribution were transformed into natural logarithm. A *p*-values less than or equal to 0.05 was considered statistically significant. All analyses were performed with SPSS® 13.0 for Windows.

The study received the approval of the local Ethics Committee of the University Hospital of Modena (prot. n. 68/13, date June 12, 2015).

## Results

Fourteen males (48.3 %) and 15 females (51.7 %) were enrolled in the study. Their mean age was 9.95 years (SD 3, min 3.72, max 17.18). Mean anthropometric measures were: weight 32.93 Kg (SD 13.76, min 15, max 64), z-weight -0.15 (SD 1.30, min -3.16, max 2.26); height 135.55 cm (SD 17.43, min 100, max 173), z-height -0.04 (SD 1.17, min -2.41, max 1.61); BMI 17.11 (SD 3.05, min 12.8, max 23.5), z-BMI -0.14 (SD 1.1, min -2.42, max 2.31). Clinical indices of disease severity were: mean number of hospital admissions per year 1.07 (SD 1.03, min 0, max 4), mean number of days of hospital admission per year 7.15 (SD 7.65, min 0, max 24), mean number of lifetime episodes of ACS 1.24 (SD 2.08, min 0, max 8). Mean hematological indices of disease severity were: total Hb 9.59 gr/dl (SD 1.71, min 5.9, max 13.7), HbF 8.13 gr/dl (SD 9.12, min 0.6, max 45.3), LDH 924.32 U/L (SD 368.92, min 468, max 1768).

Using analysis of variance, z-weight (α = 9.672, β = 0.545, r2 = 0.171, *p* = 0.026) and z-BMI (α = 9.671, β = 0.590, r2 = 0.152, *p* = 0.037) resulted significantly directly related to Hb levels. All other anthropometric measures didn’t show any significant correlation to disease severity indices.

Desired daily amount and actual daily intake of total calories, macro- and micronutrients were compared using Student’s *t*-test and resulted all significantly different. Protein, vitamin PP and A intakes were significantly over daily requirement; total caloric intake, carbohydrate, lipid, as well as all minerals (except phosphorus) and remaining vitamin intakes were significantly below daily requirement (Table 1).

**Table 1:** Comparison between daily nutrient requirement and estimated patients’ intake of calories, macronutrients, minerals and vitamins

		Daily Requirement mean ± SD	Patients’ Intake mean ± SD	*p*	% Daily intake/Daily requirement mean ± SD
		(min-max)	(min-max)		(min-max)
MACRO NUTRIENTS	Calories (Kcal)	1798 ± 301	1514 ± 325	<0.001	85.62 ± 20.43
		(1047–2403)	956–2109		51.68–141.24
	Carbs (g)	251.41 ± 39.33	220.79 ± 52.72	<0.001	88.97 ± 21.28
		(152–332)	117–337		43.33–134.87
	Protein (g)	42.38 ± 13.84	52.28 ± 14.71	<0.001	133.10 ± 49.42
		18–74	25–80		66.67–231.82
	Lipids (g)	69.21 ± 11.69	55.10 ± 11.44	<0.001	96.21 ± 29.02
		41–93	32–82		14.44–157.89
MINERALS	Ca2+ (mg)	869.59 ± 221.7	462.2 ± 240.9	<0.001	56.43 ± 34.41
		508–1293	119–1321		16.79–146.13
	Fe (mg)	10.67 ± 3.96	5.85 ± 2.20	<0.001	59.15 ± 24.99
		6–20	2.17–11.04		25.09–106.67
	P (mg)	869.59 ± 221.7	831.5 ± 246.4	<0.001	100.1 ± 35.14
		508–1293	361–1611		51.85–175.19
VITAMINS	B1 (mg)	0.81 ± 0.17	0.44 ± 0.15	<0.001	55.4 ± 17.72
		0.44–1.18	0.17–0.81		23.94–96.00
	PP (mg)	12.17 ± 2.57	23.73 ± 7.15	<0.001	198.6 ± 57.74
		7–17	13–38		117.60–330.80
	A (mcg)	470.41 ± 96.89	521.19 ± 296.7	<0.001	120.5 ± 88.28
		253–646	139–1216		29.52–425.32
	C (mg)	43.55 ± 8.48	32.05 ± 29.94	<0.001	72.9 ± 65.39
		28–64	4–113		8.45–267.64
	B2 (mg)	0.97 ± 0.23	0.91 ± 0.41	<0.001	96.7 ± 52.42
		0.50–1.51	0.31–2.22		38.75–250.00

The correlation of total calorie, macro- and micronutrient intakes, expressed as percentage of their respective daily requirements, with clinical and hematological indices of SCD severity were analyzed with analysis of variance. Using multiple regression analyses, sex and age didn’t show any influence on all the dependent variables of disease severity, so they were excluded from the statistical models. The number of hospital admissions per year was significantly inversely correlated to lipid, calcium, phosphorus and vitamin PP intakes. The days of hospital admission per year were significantly inversely correlated to protein, lipid, phosphorus, vitamins B1 and PP intakes. Levels of HbF were significantly inversely correlated to the intake of carbohydrates, lipids, iron, phosphorus, vitamins B1 and B2. All information of these correlations are reported in Table 2. Lifetime episodes of ACS, total Hb and LDH levels were not correlated to calorie, macro- and micronutrient intakes. Total calorie intake was not significantly correlated to any of the severity indices, as well as the intakes of vitamins A and C.

**Table 2:** Effects of macro- and micronutrients on indices of disease severity

	N° hospital admission/yr	N° days of hospital admission/yr	Mean HbF
Proteins	ns	α = 2.831 β =−0.009 *r* ^2^ = 0.179	ns
		*p = 0.022*	
Carbohydrates	ns	ns	α = 3.438 β =−0.019 *r* ^2^ = 0.208
			*p = 0.033*
Lipids	α = 2.425 β =−0.014 *r* ^2^ = 0.157	α = 2.931 β =−0.014 *r* ^2^ = 0.138	α = 2.998 β =−0.013 *r* ^2^ = 0.241
	*p = 0.033*	*p = 0.047*	*p = 0.020*
Calcium	α = 4.026 β =−0.762 *r* ^2^ = 0.168	ns	ns
	*p = 0.027*		
Iron	ns	ns	α = 2.600 β =−0.014 *r* ^2^ = 0.185
			*p = 0.045*
Phosphorus	α = 2.199 β =−0.011 *r* ^2^ = 0.147	α = 3.065 β =−0.015 *r* ^2^ = 0.227	α = 2.954 β =−0.012 *r* ^2^ = 0.232
	*p = 0.040*	*p = 0.009*	*p = 0.023*
Vitamin B1	ns	α = 2.911 β =−0.024 *r* ^2^ = 0.151	α = 3.023 β =−0.023 *r* ^2^ = 0.437
		*p = 0.037*	*p = 0.042*
Vitamin PP	α = 2.552 β =−0.007 *r* ^2^ = 0.174	α = 3.479 β =−0.009 *r* ^2^ = 0.255	ns
	*p = 0.024*	*p = 0.005*	
Vitamin B2	ns	ns	α = 2.459 β =−0.007 *r* ^2^ = 0.208
			*p = 0.033*

*Calories, vitamins A and C, as well as total Hb levels and lifetime episodes of ACS didn’t show any statistically significant correlation and are not reported in the table*

*ns not statistically significant*

The comparison of the last 24-h recall with weekly intake information didn’t show any statistical difference: therefore, only the information obtained from the last 24-h recall intake were reported.

## Discussion

Our study showed that body weight and BMI are significantly directly related to total Hb mean values, and thus to the severity of SCD. Several macro- and micronutrient intakes were inadequate, many of them showing a significant impact on number and days of hospital admissions. HbF, protective on SCD, on the other hand, was negatively correlated to several nutrients. To our knowledge, this is the first study that extensively evaluated the nutritional intake of macro- and micronutrients and its effect on SCD severity.

Standardized weight and BMI contributed, in our population, to the 17 and 15 % respectively of the total variance of total Hb levels. These findings are in line with others studies that reported a relationship between growth failure, and/or inadequate nutritional intake, and disease severity in SCD patients, suggesting that a targeted nutritional intervention could reduce the clinical severity of SCD [8]. Growth deficiency and undernourishment have already been described in patients with SCD, but the underlying mechanism and the precise role of potential intrinsic and extrinsic factors related to inadequate food intake or increased demands, associated with higher energy costs, are still unclear [6]. Inadequate nutritional intake, micronutrient deficiency, endocrine dysfunction and hypermetabolism have been suggested as main factors that may contribute to growth failure in children with SCD [9]. A series of study, indicated that children with SCD have a basal metabolic rate and resting energy expenditure higher than their healthy peers; this increases their metabolic, energy and protein demands. As a result, inadequate food intake found in our patients could be even more serious compared to those considered for subjects with same age, sex and anthropometric measures, but not suffering from a chronic disease [5, 10–13]. Weight and BMI didn’t show, however, any influence on other clinical and hematologic variables examined in our study, besides total Hb. This could be explained by the relatively small number of patients involved in this study and the even smaller number of children with very low weight and BMI, leading to low statistical power.

The analysis of the last 24-h recall food intake of our patients, with the software used by the Italian National Research Institute for Food and Nutrition, showed that total calorie, some macro- and micronutrient intakes were significantly inadequate when compared to daily requirements. The most deficient intakes involved calcium, iron, vitamins B1 and C. Total calorie, carbohydrate, lipid, and vitamin B2 intakes were only moderately below daily requirements, while protein and vitamins PP and A intakes in our population were high. This is because the diet of our children was mainly based on meat, canned tuna, rice and carrots. Total calorie intake didn’t show, in our population, any correlation with clinical and hematologic variables. Regarding macronutrients, proteins showed a significant negative correlation to the number of days of admission per year and carbohydrates to HbF. Lipid intake was significantly inversely related to the number and days of hospitalization, and to HbF. Heyman et al. claimed that nutritional supplementation may reduce clinical illness, and supplements administered by nasogastric tube to children with SCD and growth deficit (weight and height below the 5th percentile) have led to a rapid and substantial increase in their growth and to a reduction of painful crises and infectious episodes [8]. Wang et al., found that therapies such as transfusion therapy, splenectomy and treatment with hydroxyurea reduce disease severity or its complications, inducing an improvement in nutritional status and growth in patients with SCD [14]. Also VanderJagt et al. argued that paying more attention to food intake could improve body composition, especially regarding lean mass, having a positive impact on morbidity and mortality [5]. On the other hand, Peters et al. found that ketones generated from fasting can reactivate production of HbF; in particular B-hydroxybutyrate generated from fasting are associated with increased levels of HbF. In this way, uncontrolled lipolysis could produce B-hydroxybutyrate in such quantities as to induce a clinically significant increase in HbF levels [15]. It could be worthwhile to study the possibility of increasing the levels of HbF in patients with SCD through a particular type of diet, resulting in a potential improvement of their condition.

In our patients, calcium and phosphorus intakes were significantly inversely related to the number of hospitalizations the first, and to both number and days of hospitalizations the latter. Iron and phosphorus intakes showed a significant negative correlation to HbF levels, thereby appearing to be protective in SCD. Kawchak et al., evaluating the daily intake over the last 24 h in American children with SCD, showed a sub-optimal intake of many nutrients in all ages, including a low intake of vitamins D and E, folic acid, calcium, phosphorus, magnesium and zinc, tending to a poorer diet with increasing age, especially during adolescence [16]. Iron deficiency is also very common in subjects with SCD [17], especially among children living in developing countries where iron deficiency anemia is widespread [18]. This finding is in agreement with our study, in which almost all patients didn’t take the recommended daily amount of iron, with very consistent deficit in some cases. Iron deficiency in patients with SCD may be beneficial because it could reduce red blood cell sickling by decreasing the mean corpuscular hemoglobin concentration with consequent reduction of hemolysis [19, 20] and painful crises [21]. It has also been suggested that iron supplementation might trigger painful crises [22]. Evidences of clinical benefit of iron deficiency are still minimal and limited because of the difficulties in assessing disease severity [23]. At the same time iron deficiency is, however, associated with a reduction of physical and intellectual growth [24]. Children with iron deficiency are therefore at risk of both growth and neurocognitive delay [5].

Vitamin B1 intake significantly inversely correlated to HbF levels, contributing to the 44 % of its total variance, as vitamin B2. Vitamin B1 and PP intakes were significantly negatively correlated to the number of days of hospitalization. Vitamins A and C didn’t have any impact on clinical severity indices in our study. Other studies have shown the correlation between vitamin A deficiency and SCD clinical severity. Schall et al. showed that the serum level of vitamin A in American children with SCD was normal in 66 % of cases and inadequate in 17 % of cases and the rate of hospital admissions was higher in children with vitamin A deficiency [25]. In our study, we didn’t find any significant correlation between the intake of vitamin A and disease severity. This can be explained by the evidence that our patients took, on average, higher amounts of vitamin A than the recommended daily requirement. An insufficient intake of vitamin C in SCD subjects has already been reported in the literature [26].

Current SCD disease treatment guidelines only underline the importance of specific micronutrients (folic acid, zinc, iron) for their involvement in inflammatory status and immunity, but make no reference to the importance of maintaining an adequate complete nutritional intake [1, 27–29].

In our study, we clearly showed that body weight and BMI can affect SCD severity, as well as inadequate specific nutrient intakes. An unexpected result was the beneficial effect that low intakes of several macro- and micronutrients can have on HbF level, known to be protective against SCD complications. Weaknesses of this study were the small number of enrolled subjects and using data derived from simple interviews with patients regarding their nutritional habits, therefore potentially not as reliable as measurement of serum levels.

## Conclusions

In this study we showed that low body weight and BMI can negatively affect total Hb levels, one of the most important parameters to define SCD severity. This despite the small number of subjects enrolled in the study. Although specific macro- and micronutrient low intakes can have a significant negative impact on disease severity assessed through number and days of hospital admissions, they also seem to potentially protect patients through an increase in HbF levels.

This study also suggests the importance of regular nutritional and anthropometric assessments in SCD children not indicated in current SCD treatment guidelines.

## Abbreviations

ACS: acute chest syndrome
BMI: body mass index
Hb: total hemoglobin
HbF: hemoglobin F
LDH: lactate dehydrogenase
SCD: sickle cell disease
SD: standard deviation
Z: standardized scores
